# MYC-Mediated Inhibition of ARNT2 Uncovers a Key Tumor Suppressor in Glioblastoma

**DOI:** 10.21203/rs.3.rs-4810280/v1

**Published:** 2024-08-15

**Authors:** Yi-Heng Hao, Nofit Borenstein-Auerbach, Anthony Grichuk, Li Li, M. Carmen Lafita-Navarro, Shun Fang, Pedro Nogueira, Jiwoong Kim, Lin Xu, Jerry W. Shay, Maralice Conacci-Sorrell

## Abstract

Tumor initiation and progression rely on intricate cellular pathways that promote proliferation while suppressing differentiation, yet the importance of pathways inhibiting differentiation in cancer remains incompletely understood. Here, we reveal a novel mechanism centered on the repression of the neuronal-specific transcription factor ARNT2 by the MYC oncogene that governs the balance between proliferation and differentiation. We found that MYC coordinates the transcriptional repression of ARNT2 through the activity of polycomb repressive complex 2 (PRC2). Notably, ARNT2, highly and specifically expressed in the central nervous system, is diminished in glioblastoma, inversely correlating with patient survival. Utilizing in vitro and in vivo models, we demonstrate that ARNT2 knockout (KO) exerts no discernible effect on the in vitro proliferation of glioblastoma cells, but significantly enhances the growth of glioblastoma cells in vivo. Conversely, ARNT2 overexpression severely dampens the growth of fully transformed glioblastoma cells subcutaneously or orthotopically xenografted in mice. Mechanistically, ARNT2 depletion diminishes differentiation and enhances stemness of glioblastoma cells. Our findings provide new insights into the complex mechanisms used by oncogenes to limit differentiation in cancer cells and define ARNT2 as a tumor suppressor in glioblastoma.

## Introduction

To grow indefinitely, cancer cells often lose their ability to differentiate and acquire stem-like properties [15]. Multiple studies have shown that the universal oncogene and transcriptional factor MYC inhibits terminal differentiation in multiple cell types, including muscle cells, and B lymphocytes [1, 9, 28, 37, 48]. Traditionally, MYC is believed to inhibit differentiation by regulating the expression of its target genes, miRNAs, and lincRNAs [31] that may prevent cells from exiting the cell cycle and undergoing terminal differentiation [41]. The role of MYC in inhibiting differentiation has important implications for cancer, as MYC is upregulated in nearly all human tumors [11]. As a broad-acting transcription factor MYC regulates the expression of numerous genes, some directly via binding as a heterodimeric complex with MAX to E-Box motifs in their promoters [7], and others by allowing amplification of genes already poised to be expressed [10, 34]. While MYC is primarily a transcriptional activator, it can also indirectly repress gene expression through less established mechanisms. For example, loss of MAX leads to substantial upregulation of many genes, which are often repressed by MXD/MNT and possibly MGA [32]. Moreover, MYC can drive gene repression through the transcriptional induction of repressors such as EZH2 [22].

MYC is elevated in 60–80% glioblastoma (GBM), and MYC expression correlates with glioma grade [19, 35]. Transgenic expression of MYC in mouse astrocytic lineages is sufficient to cause gliomas that resemble the human disease [21]. Activation of MYC downstream of *p53* and *Pten* mutations is associated with impaired neuronal differentiation and enhanced self-renewal capacity of GBM cells [52]. Importantly, MYC has been shown to play a critical role in GBM progression in which MYC regulates genes involved in cell cycle, metabolism, and differentiation [3]. Although the roles of MYC in inducing genes that promote cell growth and metabolic reprogramming are well understood, the mechanisms through which MYC restricts differentiation remain less clear.

Here, we report that MYC represses the expression of A the bHLH-PAS (basic helix-loop-helix-Per/ARNT/Sim) transcription factor Aryl Hydrocarbon Receptor Nuclear Translocator 2 (ARNT2). ARNT2 is directly involved in neural development, particularly in the formation and patterning of the cerebral cortex, hippocampus, olfactory bulb, cerebellum, retina, and inner ear [20, 23, 49]. ARNT2 regulates genes involved in neuronal differentiation, migration, and survival, thereby playing a crucial role in the development and function of the central nervous system [5, 40] and dysregulation of ARNT2 expression or function has been implicated in several neurodevelopmental disorders, including autism spectrum disorder and schizophrenia [49]. We found that MYC represses ARNT2 transcription indirectly in a mechanism requiring the polycomb complex proteins SUZ12 and EZH1/2. We demonstrated that ARNT2 expression, which is exclusively confined to the central nervous system, is lost in high-grade glioma. Knocking out ARNT2 in cells leads to inhibition of differentiation programs and increase of stem markers. Moreover, loss of ARNT2 leads to an increase of GBM cell proliferation in vivo and ARNT2 overexpression prevents it, implicating ARNT2 may act as a tumor suppressor in GBM.

### Results

## Repression of ARNT2 expression by MYC is mediated by polycomb repressive complex 2

We previously demonstrated that MYC activates the expression of the growth-promoting bHLH-PAS domain transcription factors aryl hydrocarbon receptor (AHR) and ARNT [29, 30]. To examine the importance of MYC in broadly regulating the expression of PAS domain transcription factors, we examined an RNA-seq dataset of Rat1 *myc-/-* fibroblasts expressing empty vector or reconstituted with human MYC [29], and found that MYC expression not only activated the bHLH-PAS domain transcription factors AHR, ARNT, and AHRR, but also suppressed several members of the same family, including neuronal PAS domain protein 4 (NPAS4), single-minded homolog 2 (SIM2), and ARNT2 (Fig. 1A and Figure S1A). ARNT2 recruits NPAS4 in response to increased neuronal activity, and this dimer induces transcription and increases somatic inhibitory input [39].

Our lab has extensively investigated the importance of the PAS domain transcription factors AHR and ARNT as growth-promoting genes downstream of MYC [29]. In the current study, we focused on the repression of ARNT2 in MYC-transformed cells. We confirmed that human MYC introduction suppressed ARNT2 expression in *myc-/-* Rat1 fibroblasts and activated AHR and ARNT expression, as demonstrated by using Western blots (Fig. 1B and Figure S1B) and RT-qPCR (Fig. 1C). Immunofluorescence (IF) analysis further revealed that MYC expression reduced nuclear ARNT2 levels while increasing ARNT expression (Fig. 1D). Expression of the cytosolic MYC fragment MYC-nick that lacks DNA binding domain [9] did not affect ARNT2 expression (Fig. 1C and 1E). Conversely, knocking down MYC in Rat1 fibroblasts augmented ARNT2 protein levels (Fig. 1F). We confirmed that ARNT2 protein and mRNA were repressed in other cell lines including the human retinal pigment epithelial cell line ARPE-19 (Fig. 1G) and that human colon cancer cell line DLD1 (Fig. 1H and 1I) that had elevated MYC. Our results show that MYC undoubtedly downregulates the expression of ARNT2.

To determine the mechanisms by which MYC causes transcriptional repression of ARNT2, we explored multiple MYC-dependent repression pathways. We asked whether the MYC-activated heterodimers AHR-ARNT and AHRR-ARNT [29] function as feedback modulators by repressing ARNT2 (Figure S1C). Knocking down AHR and AHRR, a transcriptional repressor regulated by AHR, had no effect on ARNT2 expression (Figure S1D-E). We also tested whether ARNT2 repression was mediated by the MAX-MNT-MLX network, which has been reported to downregulate gene expression by binding to E-boxes [12] (Figure S1F). We determined that knocking down MAX, MLX, and MNT also had no effect on ARNT2 levels (Figure S1G). Finally, we tested the involvement of the polycomb repressive complex 2 (PRC2) in MYC-regulated ARNT2 repression. The PRC2 complex is composed of SUZ12, EED, RBBP4, and histone methyltransferase EZH2 or EZH1, and it represses gene expression by methylating lysine 27 of histone H3 [47]. MYC had been previously implicated in transcriptional repression via the activation of the methyltransferase subunit EZH2 [22]. Interestingly, we found MYC induces the expression of Ezh2 in Rat1 *myc*-/- fibroblasts as determined by RNA-seq (Figure S1H). To investigate whether PRC2 regulates ARNT2 expression, we knocked down EZH2 and its homologue EZH1 in ARPE-19 cells expressing empty vector or MYC (hereafter referred to as ARPE and ARPE MYC, respectively). We observed that MYC expression upregulated both EZH2 and EZH1 expression, and reciprocal knockdown experiments showed that reducing either EZH2 or EZH1 increased the other (Fig. 1J). Consequently, we performed double knockdowns of both genes. Knocking down EZH2 or EZH1 individually in ARPE cells increased ARNT2 expression, whereas only EZH2 knockdown in ARPE MYC cells elevated ARNT2 protein levels (Fig. 1J).

To further investigate PRC2's role, we knocked down SUZ12, another member of the complex, in both cell types. SUZ12 levels were elevated in MYC-overexpressing cells, and its knockdown substantially decreased EZH2 and EZH1, likely due to complex destabilization (Fig. 1K). Crucially, SUZ12 knockdown also increased ARNT2 levels in both cell lines (Fig. 1K). Subsequently, we used two histone methyltransferase inhibitors: UNC1999, which targets both EZH2 and EZH1 activities, and GSK126, which is a highly selective EZH2 inhibitor [51]. We observed that the inhibitors reduced methylation levels of lysine 27 on histone H3 in both cell types, with a more pronounced increase in ARNT2 levels in ARPE MYC cells than in ARPE cells. Specifically, only UNC1999 in ARPE cells showed a slight increase in ARNT2 levels (Fig. 1L). These findings led us to conclude that MYC upregulates EZH2 expression, thereby enhancing methylation of lysine 27 on histone H3 and consequently repressing ARNT2 expression (Fig. 1M). Supporting this, our analysis of the ENCODE database revealed H3K27Me3 and EZH2 peaks at the ARNT2 promoter in multiple experiments (Figure S1I-J).

## ARNT2 is highly expressed in brain and cerebellum and repressed in GBM

Given that MYC is a proto-oncogene, which regulates the initiation and progression of many human cancers [10], we investigated the tumor types for which repression of ARNT2 by MYC may be the most biologically meaningful. Comparing ARNT2 mRNA levels in tumor and normal tissues from the TCGA and GTEx database, we found that ARNT2 levels were elevated in several cancer types including breast invasive carcinoma (BRCA), pancreatic adenocarcinoma (PAAD), and skin cutaneous melanoma (SKCM) (Figure

S2A). In contrast, ARNT2 levels were repressed in colon adenocarcinoma (COAD), kidney renal clear cell carcinoma (KIRC), and most importantly, GBM (Fig. 2A). To examine the relationships between patient survival and ARNT2 expression, we used the Xena Functional Genomics Explorer that links TCGA survival data to gene expression level and found that ARNT2 expression was inversely correlated with poor patient survival in two types of cancers, PAAD and glioma (LGG-GBM) (Fig. 2B and S2B). We also observed that ARNT2 levels had no significant impact on survival in patients with GBM, likely attributed to the aggressive nature of this disease (Figure S2B). TCGA data revealed a decrease in ARNT2 levels with higher glioma grades (Fig. 2C), a trend corroborated by analysis of the Chinese Glioma Genome Atlas (CGGA) (Figure S2I).

Using GTEx analysis of human tissue gene expression profiles, we identified specific ARNT2 mRNA expression in the brain including the cerebellum (Figure S2C), which was further validated by Western blotting of mouse tissue samples (Fig. 2D). Given this distinct expression pattern of ARNT2 in normal brain and cerebellum, coupled with bioinformatics analyses indicating its reduced presence in glioblastoma, we directed our investigations towards understanding the role and regulation of ARNT2 in glioblastoma.

By comparing ARNT2 levels in glioblastoma cell lines by Western blotting, we found that ARNT2 expression was highest in LN229 cells, followed by U87 cells, and was not detected in GBM9 and SF188 cells when compared to astrocytes (Fig. 2E). As expected for a MYC-repressed gene, ARNT2 and MYC levels were inversely correlated. Knocking down MYC in LN229 cells increased ARNT2 levels even higher and reduced ARNT levels (Fig. 2F). Knocking down AHR and AHRR did not alter ARNT2 expression which was consistent with the results in ARPE cells (Figure S2D-E). Knocking down MAX and MNT in LN229 had no consistent effects on ARNT2 expression in GBM cells (Figure S2F). However, knocking down EZH2 and EZH1 using siRNAs in LN229 and U87 cells led to an increase in ARNT2 expression when compared with samples transfected with control siRNA (Fig. 2G). The changes were more dramatic in U87 cells, which have lower ARNT2 expression compared to LN229, and with siRNA of EZH2, which is the primary methyltransferase in PRC2. Similar to ARPE and ARPE MYC cells, knocking down SUZ12 also increased ARNT2 in both LN229 and U87 cells (Fig. 2H), and inhibiting methyltransferase activities by UNC1999 and GSK16 had similar effects (Fig. 2I). Furthermore, knocking down PRC2 members from SF188 cells also increased the ARNT2 levels although ARNT2 levels in these cells are extremely low (Figure S2G). Thus, we confirmed that PRC2 represses ARNT2 expression in GBM cells. Xena Functional Genomics Explorer revealed that higher EZH2, but not EZH1, correlated to shorter survival of glioma patients (Figure S2H). Both TCGA and CGGA data revealed that the levels of EZH2 and SUZ12 are higher in high-grade glioma (Fig. 2J and S2I).

#### Knocking out ARNT2 decreases the expression of neuronal markers and increases the expression of stem cell markers in GBM cells

To begin investigating the importance of ARNT2 in GBM biology, we first confirmed that ARNT2 localized to both cytosolic and nuclear fractions in both LN229 and U87 cells (Fig. 3A). We used LN229 cells, which express the highest levels of ARNT2, to investigate the effects of ARNT2 loss in GBM cells. We knocked out ARNT2 from LN229 cells by CRISPR and selected single clones (Fig. 3B). Two clones (B3 and C4) along with control LN229 cells were subjected to RNA-seq analysis that identified about 600 genes with at least

50% expression level changes in both clones compared to control cells ($p < 0.05$) (Figure S3A-B). Gene Ontology (GO) analysis revealed that these genes were highly enriched in organism and nervous system development, cellular response to stimulation, and cell and neuron differentiation (Fig. 3C, Figure S3C-D).

Among the genes upregulated upon ARNT2 knockout (KO), some exhibit increased expression levels with higher glioma grades, which also correlate with poorer patient survival (Figure S3C). These include genes involve in cellular signaling such as regulator of G protein signaling 16 (RGS16) and interleukin 13 receptor subunit alpha 2 (IL13RA2), cell differentiation genes such as ectopic viral integration site 2B (EVI2B), and the innate immune response genes to viral infection like 2'-5'-oligoadenylate synthetase 2 (OAS2). Among genes downregulated upon ARNT2 KO, some were repressed in the higher glioma grades, likely resulting in better patient prognosis (Figure S3D). These include genes involve in neuronal functions like potassium calcium-activated channel subfamily N member 2 (KCNN2) and SLIT and NTRK like family member 4 (SLITRK4), gene expression regulation such as Scm like with four Mbt domains 2 (SFMBT2), and abnormal immune response genes to tumor such as PNMA family member 5 (PNMA5).

Based on GO analysis and the ARNT2 expression profile (Fig. 2D), we investigated the roles of ARNT2 in neuronal differentiation of GBM cells. Cultured GBM cells contain a heterogeneous population of cells, including cancer stem cells, which means they can be differentiated into a phenotypically diverse cell population, including neurons [13]. ARNT2 KO cells cultured in medium containing low serum had a higher expression of the stem cell marker Nestin but lower levels of the astrocyte marker GFAP and the neuronal marker acetylated tubulin (Fig. 3D), suggesting that without ARNT2, LN229 cells grown in low serum were more stem-like and less differentiated. Knocking out ARNT2 led to increase of neuronal marker acetylated tubulin in LN229 cells grown as suspended spheres and induced differentiate by serum starvation (Figure S3E). The expression of cell cycle and growth regulators were also increased upon ARNT2 KO including cyclin A1 and phosphorylated p70 S6 kinase and mTOR (Fig. 3D), indicating loss of ARNT2 may facilitate proliferation. By examining the expression of genes involved in neuronal differentiation identified in RNA-seq (Fig. 3C), we found that their expression was lower in high-grade gliomas (Fig. 3E). Similar to ARNT2, lower expression of these genes correlated with shorter survival of glioma patients (Fig. 3F).

To ascertain the importance of ARNT2 in neuronal differentiation, we utilized a human induced pluripotent stem cells (iPSCs) model expressing a doxycycline-inducible master neuronal transcriptional regulator neurogenin-2 [14]. Neurogenin-2 induction resulted in the differentiation of iPSCs into neurons in three days. By comparing the Western blots of iPSC samples before and after differentiation, we found that ARNT2 was greatly elevated in differentiated neurons, similar to the neuronal differentiation markers acetylated-tubulin, β3-tubulin, and UCHL1 (Fig. 3G), indicating high ARNT2 expression is associated with neuronal differentiation. Knocking down ARNT2 by siRNAs in iPSCs cultured in the presence of doxycycline decreased level of acetylated-tubulin, and to a lesser extent, levels of β3-tubulin and UCHL1 (Fig. 3G), confirming the importance of ARNT2 in neuronal differentiation.

### Knocking out ARNT2 promotes the growth of GBM cells in vivo

Given our initial observations suggesting that loss of ARNT2 reduces cell differentiation and promotes stemness, we investigated its impact on cell proliferation. Our results demonstrate that ARNT2 KO in LN229 cells did not significantly alter cell proliferation in vitro (Fig. 4A). Similarly, knocking down ARNT2 transiently did not globally affect proliferation of ARPE, ARPE MYC, DLD1, LN229, U87, and GBM9 cells transfected with up to 4 ARNT2 siRNAs (Figure S4A-E, and Figure S4F confirms ARNT2 knockdown in DLD1 cells). Nevertheless, despite these in vitro findings, loss of ARNT2 had notable effects on GBM cell growth in vivo when into NOD scid mice (Fig. 4B). We observed that tumors from ARNT2 KO clone B3 were more than double the weight of tumors from WT cells, whereas tumors from clone C4 were less affected (Fig. 4C). Western blot analysis of neuronal marker UCHL1 in tumor lysates revealed lower levels in clone B3 and C4 tumors than in WT tumors, with clone B3 tumors showing the lowest UCHL1 levels, correlating with smaller tumor size (Fig. 4D).

By performing hematoxylin and eosin (H&E) staining of paraffin-embedded tumors shown in Fig. 4D, we found that the only visible difference between tumors formed from control cells and ARNT2 KO cells was the presence of lipid droplets in the ARNT2 KO tumors (Fig. 4E and S4G). Recent studies have suggested that increased in lipid droplets in human tumor tissues, particularly GBM, is correlated with increased disease aggressiveness [16]. In agreement, metabolomics analyses found that tumors derived from ARNT2 KO cells displayed a dramatic increase in lipids and to a lesser extend nucleotides. Among the lipids upregulated in ARNT2 KO tumors were two carnitines associated with long-chain fatty acids, which can be used to generate energy for tumor growth via b-oxidation. Furthermore, metabolomics analyses revealed dramatic increases in the levels of phospholipids upon ARNT2 KO. These include 11 phosphatidylcholines (PC) out of 13 readable in the metabolomics platform, 3 out of 7 phosphatidylethanolamines (PE), 1 out of 4 phosphatidylserines (PS), and 2 out of 4 sphingomyelins (SM). PC and PE are the most abundant phospholipids in all membranes of mammalian cells, including the monolayer membrane surrounding lipid droplets. It has been reported that PC and PE regulate the size and dynamics of lipid droplets, and the differentiation of adipocytes [46]. PS is the major anionic phospholipid of brain, and it is particularly enriched in the cytoplasmic leaflet in the plasma membrane, functioning as an anionic domain that binds and thereby activates cytosolic neuronal signaling [24]. SM is highly enriched in the membranous myelin sheath that surrounds axons of neurons, as well as the outer leaflet of plasma membrane. Increase in outer leaflet SM content has been connected with cancer initiation, growth, and immune evasion [44]. In agreement with the increase in phospholipids, the expressions of phospholipases, including several members of phospholipase A2 (except PLA2G4C), phospholipase D3 (PLD3), and a predicted phospholipase B (PLBD1), were lower with ARNT2 KO in our RNA-seq dataset (Figure S5A). Phospholipases cleave ester bonds within phospholipids to generate fatty acids and other lipophilic substances [2]. Phospholipase A2, which hydrolyzes the sn-2 acyl chain of phospholipids to generate free polyunsaturated fatty acids, is considered as the key enzyme and plays important roles in inflammation [4] and lipid droplet formation [17]. In addition to decreasing the expression of phospholipases, ARNT2 KO increased the expression level of Annexin A1 (ANXA1), which binds phospholipids and inhibits phospholipase A2 [25]. We also found that ARNT2 regulated the expression of other genes involve in lipid metabolism, including fatty acid desaturase (FADS2), which is essential to maintain stem cells state glioblastoma cells [38], acyl-CoA synthetase 5 (ACSL5) that activate fatty acids (FAs) by thioesterification with Coenzyme A, Perilipin 4 (PPLIN4), which coats lipid droplets protecting them in the cytoplasm, and the master regulator of adipocyte differentiation (PPARG) (Figure S5B). On the basis of these metabolomics data and RNA-seq analysis, we propose that ARNT2 KO promotes lipid biosynthesis to sustain growth.

## Ectopic expression of ARNT2 blocks the growth of subcutaneously xenografted GBM cells

The increase in GBM tumor growth upon ARNT2 KO prompted us to investigate the potential role of ARNT2 as a tumor suppressor in GBM. Thus, we generated LN229 cells ectopically expressing empty vector or GFP-tagged ARNT2 (Fig. 5A). Similar to endogenous ARNT2, GFP-ARNT2 also localized to the nucleus, thus indicating that this tagged protein is functional (Fig. 5B). Ectopic expression of ARNT2 did not affect the growth of LN229 cells in vitro (Fig. 5C), thus we performed xenotransplantation experiments (Fig. 5D). We found that tumors derived from cells overexpressing ARNT2 were significantly smaller than those from cells empty vector, measured eight weeks after transplantation into NOD scid mice (Fig. 5E-F and Figure S5C). Western blots of protein lysates from excised tumors found elevated UCHL1 levels in ARNT2-overexpressing tumors, which supports our data from the ARNT2 KO experiments (Fig. 5G). To extend our findings to additional GBM cells, we generated GBM9 cells expressing either empty vector or ARNT2 (Fig. 6A) and found modest difference in the in vitro growth of these cells (Fig. 6B). However, xenograft tumors (Fig. 6C) from cells expressing ARNT2 were significantly smaller than those from cells expressing empty vector (Fig. 6D-E and Figure S6A). Western blots of protein lysates from excised tumors demonstrated slightly higher UCHL1 and a reduction of synaptophysin, a marker for neuroendocrine tumors [33], in ARNT2 expressing tumors (Fig. 6F).

## Ectopic expression of ARNT2 reduced the growth of orthotopically xenografted GBM cells

To better understand the importance of ARNT2 loss in glioblastoma physiology, we performed orthotopic engraftment of empty vector and ARNT2-expressing GBM9 cells into the brain of NOD scid mice (Fig. 6G). This experiment revealed that empty vector-expressing cells formed significantly larger tumors compared to ARNT2-expressing cells. Tumors are distinguishable in dissected brains because of their GFP signal (Fig. 6H). H&E staining confirmed that tumors arising from vector-expressing cells are larger than the ones arising from ARNT2-expressing cells (Fig. 6I). The overall morphology of GBM9 empty vector and ARNT2 expressing cells in the tumors was similar (Figure S6B). Importantly, we found that mice injected with ARNT2-expressing cells survived longer than those injected with empty vector-expressing cells (Fig. 6J). As expected, Nestin and synaptophysin were lower in ARNT2-expressing tumors compared to empty vector-expressing cells (Fig. 6K). Comparing the lysates of tumors with those of cells in culture, we found that the change of Nestin is specific to tumors (Figure S6C). MYC-nick, the cytosolic MYC variant associate with terminally differentiated tissues [9], was accumulated in ARNT2-expressing tumors (Fig. 6K).

### Discussion

We propose that MYC promotes GBM growth by suppressing ARNT2 expression through the induction of polycomb genes. MYC has been shown to promote dedifferentiation and stemness in GBM [43] and repression of ARNT2 is likely critical for this process. Interestingly, a similar mechanistic model has been proposed for prostate cancer, where MYC prevents differentiation by upregulating EZH2 [26]. The global regulation of bHLH-PAS transcription factors by MYC plays a vital role in cellular functions [6]. For example, MYC-mediated deregulation of the circadian clock genes, CLOCK and BMAL1, affects growth specialization in various tissues. Importantly, disruption of the circadian clock by MYC impairs cellular differentiation and promotes tumorigenesis [45]. We now show that ARNT2, believed to be closely related to ARNT, is repressed by MYC. Studies by others showed that GBM requires large amount of lipids for rapid growth, and that substantial quantities of lipids are stored as the form of lipid droplets, which are undetectable in normal brain tissues [27, 42]. Lipid droplets play critical roles in GBM growth and survival [50], which we believed to be at least in part, regulated by ARNT2.

The role of ARNT2 in cancer is controversial and appears to be tissue specific. For example, ARNT2 is upregulated in human colorectal cancer (CRC), and that knockdown of ARNT2 inhibits CRC cell proliferation and invasion in vitro as well as tumor growth and metastasis (Wang et al., 2018) through the activation of the Wnt/β-catenin signaling pathway. The MYC-induced gene ARNT is closely related to ARNT2 sharing 61% overall sequence identity, including 12 of the 13 amino acids in the basic regions, and 80% identity of HLH and PAS regions. While ARNT is expressed ubiquitously, ARNT2 is expressed predominantly in brain, and is associated with neuroprotection. During neuronal differentiation, ARNT2 expression increases while ARNT mRNA levels decreases [18], thus further demonstrating that these factors are not functionally similar. Similar to GBM, ARNT2 expression is also lower in human breast cancer tissues, and that overexpression of ARNT2 inhibits breast cancer cell proliferation, migration, and invasion in vitro and tumor growth and metastasis in vivo. Another study suggested that repression of ARNT2 was associated with loss of GBM cell tumorigenicity; however, only limited experimentation was performed to test this model [8]. We demonstrate that ARNT2, by promoting cell differentiation, functions as a tumor suppressor in GBM and possibly other tumors from neuronal origin. ARNT2 loss leads to tumor growth, and its upregulation prevents it. This role as a tumor suppressor likely rests on its ability to drive the expression of genes that define the commitment to neuronal differentiation, thus ARNT2 loss may drive stemness.

## Declarations

### ACKNOWLEGEMENT

We are grateful to the Sorrell lab members for their valuable feedback. The research was financially supported by Cancer Prevention and Research Institute of Texas (CPRIT) (RP220046), American Cancer Society (724003), Welch Foundation (I-2058-20210327), NCI R01CA245548, NIGMS GM145744-01 to MCS, U19CA264385 to JWS, Mary Kay postdoctoral fellowships to PN and SF, Mechanisms of Disease and Translational Science T32 to AG. MCS is the Virginia Murchison Linthicum Scholar in Medical Research. The authors acknowledge the support of UT Southwestern Metabolic Phenotyping Core and Histo Pathology Core.

#### Author Contributions

MCS and YHH planned the experiments and wrote the manuscript. YHH performed most of the experiments. LL, MCLN and NBA performed experiments. PN prepared tumors for IHC and SF performed image analyses. AG performed orthotopic transplantation. JK and LX performed RNA-seq data analyses. JWS advised, edited the manuscript, and provided reagents.

#### Declaration of interests

Authors have no conflicts to declare.

## Methods

#### Sex as a Biological Variable

We used female NOD scid mice for our xenograft experiments. Our previous experience demonstrated that sex has no effects on these experiments.

#### Cell Cultures

Rat1 fibroblasts, human epithelial cell ARPE-19, human colon cancer cell DLD1, and human glioblastoma cells GBM9, SF188, U87 and LN229 were cultured in DMEM with 5% FBS, 100 U/mL pen/strep. For suspension culture, LN229 cells were cultured in DMEM with 5% FBS, 100 U/mL pen/strep on plate coated with poly-HEMA. To induce differentiation, spheres were cultured in DMEM/F12 + B27 + 0.5 ml/ml BSA + 20 ng/ml EGF + 20 ng/ml FGFb for 10 days. Human-induced pluripotent stem cells (iPSCs) with doxycycline-inducible neurogenin-2 (NGN2), gifted from Dr. Erik Ullian (UCSF), were cultured in Essential 8 (E8) medium. Neuronal differentiation was induced using induction medium (DMEM/F12, HEPES + N2 Supplement + Non-essential Amino Acids + L-Glutamine + 2 mg/ml doxycycline) for 3 days [14]. siRNA was reverse transfected to cells with Lipofectamine RNAiMAX reagent following the standard protocol (Invitrogen). Briefly, on the day of transfection, $2.5 \times 10^5$ cells were seeded per well in 6-well plates and transfected with 5 μL siRNA (20 μM) and 5 μL Lipofectamine previously mixed in Opti-MEM for 15 min. After 72 h of transfection, cells were collected and analyzed.

#### Stable cell lines

ARNT2 knockout (KO) cells were generated by transfecting ARNT2 CRISPR/Cas9 KO Plasmid (Santa Cruz sc-403101) into LN229 cells with Lipofectamine LTX&Plus reagent. Three days after transfection, GFP-positive cells were sorted by flow cytometry and single clones were selected to verify the knockout by using Western blots. For cell lines stably expressing ARNT2, the *ARNT2* gene was constructed into pIRESpuro vector with N-terminal GFP tag, and the construct, together with the empty vector, were transfected into LN229 cells. The cells that stably expressing these constructs were selected with 5 mg/mL puromycin. The *ARNT2* gene was also constructed into pIRESneo vector with no tag. The construct, together with the empty vector, were transfected into GBM9 cells. The cells stably expressing these constructs were selected with 1 mg/mL G418.

#### Cell Viability Assays

Cell proliferation was measured by either crystal violet staining or Cell Counting Kit-8 (CCK-8). For crystal violet staining, the cells were cultured in 6-well or 12-well plates for 3-7 days before washed with PBS, and then fixed with methanol at room temperature (RT) for 10 min. Cells were stained with crystal violet solution containing 1% acetic acid, 1% methanol, 1% (w:v) crystal violet dye for 10 min with agitation. After washing extensively, the plates were scanned and the intensity of the signal was quantitated by using ImageJ. Results are presented to reflect the relative growth after normalization by the control condition. Experiments were repeated at least three times.

For analysis via Cell Counting Kit-8 (CCK-8), the cells were cultured in 96-well plates for 3-7 days. After the addition of 10 mL of CCK-8 reagent, the cells were put back into the incubator for 1 h and absorbance was read at 450 nm. Results are presented to reflect the relative growth after normalization by the control condition. Experiments were repeated at least three times.

#### Immunofluorescence Staining and Microscopic Imaging

Cells grown on glass plates were fixed with 4% paraformaldehyde in PBS for 15 min at room temperature (RT), and permeabilized in blocking buffer (0.1% Saponin, 3% BSA in PBS) for 20 min at 4°C. Primary antibodies in blocking buffer were added and incubated 1 hour at RT. Cells were washed 3 times with 0.1% Saponin in PBS and incubated with secondary antibodies in blocking buffer for 1 hour at RT. DAPI was used to stain the nuclei. Images were acquired with a Zeiss LSM780 microscope.

#### Western Blots of Total Cell Lysates and Nuclear/cytoplasmic Fractionation

Total protein was extracted with lysis buffer containing 10 mM Tris-HCl (pH 7.7), 50 mM NaCl, 0.5% Nonidet P-40, proteinase inhibitor cocktail (SIGMAFAST™ Protease Inhibitor Cocktail Tablets, EDTA-Free), and 1 mM DTT. For fractionation, the cells were extracted with Buffer A (10 mM HEPES-KOH pH 7.9, 10 mM KCl, 1.5 mM $MgCl_2$, 0.5% Nonidet P-40) plus proteinase inhibitor cocktail and 1 mM DTT by vortex. The samples were spun at 4,000 rpm for 4 min, and the supernatants were collected as cytoplasmic fractions. The pellets were washed twice with Buffer A and then extracted with Buffer B (20 mM HEPES-KOH pH 7.9, 400 mM NaCl, 1.5 mM $MgCl_2$, 0.2 mM EDTA, 15% glycerol) plus proteinase inhibitor cocktail and 1 mM DTT with sonication. The samples were spun at 15,000 rpm for 10 min, and the supernatants were collected as nuclear fraction. Protein concentrations were measured by using the Bradford protein assay, and the samples were normalized to same protein concentration. Proteins were separated by SDS–polyacrylamide gel electrophoresis, and then transferred to nitrocellulose membranes (Thermo Fisher), probed with specific antibodies, and detected by using chemiluminescence with the Bio-Rad Image lab.

#### RNA-seq

WT and ANRT2 KO LN229 cells were collected and sent to GENEWIZ for RNA extraction and sequencing. RNA-seq assessment of the raw sequencing reads was done using the NGS-QC-Toolkit [36]. The reads were aligned to the genome RGSC 6.0/rn6 using HISAT2 (v 2.1.0) aligner. A minimum read count filter of 10 total reads was applied to remove low-expressed genes. Filtered reads were then normalized using DESeq2. DeSeq2 employs a negative binomial distribution to estimate data variability and uses an error model for a more robust statistical test for significance. An FDR cutoff of <5% was used to select significantly altered genes between experiment conditions. Gene Ontology analysis was performed with GO Consortium (http://geneontology.org/). RNA-seq data has been deposited in GEO with ID GSE236486.

#### RT-qPCR

RNA was extracted from cells with QIAGEN RNeasy Mini Kit and reverse transcribed to cDNA with High-Capacity cDNA Reverse Transcription Kit (Thermo Fisher). Relative RNA levels were measured by qPCR with iTaq™ Universal SYBR® Green Supermix (Bio-Rad) and the Bio-Rad CFX96 device and normalized to 18S rRNA gene.

#### Bioinformatics Analysis

ARNT2 mRNA expression in tumor and normal tissues from patients of different tumor types deposited in TCGA and GTEx was analyzed by Gene Expression Profiling Interactive Analysis (GEPIA) web server. |Log2FC| cutoff 0.585, and p-value cutoff 0.01. Kaplan-Meier curve comparing survival of cancer patients with the 25% highest and the 25% lowest levels of certain genes were generated using Xena Functional Genomics Explorer (Xena Functional Genomics Explorer). Gene expression levels in glioma grades were from TCGA and Chinese Glioma Genome Atlas (CGGA) (http://www.cgga.org.cn/).

#### Subcutaneous Xenografts

LN229 or GBM9 cells suspended in 30% Matrigel were injected into the flank of each female NOD scid mouse (Jackson lab). Mice were sacrificed when their largest tumors reached ~2 cm. At the end of the experiment, tumors were harvested, weighed, and either snap frozen in liquid nitrogen for protein extraction or fixed with 10% formalin for histology. All procedures are approved by IACUC at UT Southwestern Medical Center.

#### Orthotopic Xenograft

GBM9 cells suspended in HBSS (Ca-, Mg-) were injected orthotopically into the striatum of 9 weeks old NOD scid mice using the following coordinates (AP, 0 mm; ML, –2.5 mm; DV, –3 mm) using bregma as the starting cranial landmark (Note: While the injection took place at a depth of -3 mm in the dorsal/ventricle plane cells were injected at -2.5 mm in the dorsal/ventricle plane to allow for a 0.5 mm gap for cells to accumulate in.). Cells were injected at a rate of 1uL/20 seconds over one minute, followed by a 30-second hold before removing the syringe. All injections were facilitated through the use of a stereotactic frame. Mice were monitored weekly using bioluminescent imaging to confirm tumor engraftment/progression. Mice were euthanized based on co-morbidities, including weight, seizures, hunched back, matted fur, and head swelling.

#### Histology

Tumor tissues from xenografts experiments were fixed with 10% neutral buffered formalin for 2 days and then transferred to 70% ethanol before hematoxylin and eosin (H&E) staining performed by the UTSW tissue core.

#### Metabolomics Analyses

20-25 mg xenograft tumor tissues were homogenized and extracted with 300 ml of ice-cold methanol/water 80:20 (vol/vol). Dry samples equivalent to 10 mg of protein with SpeedVac. Qualitative assessment of global metabolites by mass spectrometry were performed by the metabolomics facility at the Children's Research Institute (UT Southwestern Medical Center, UTSW). Using a cutoff of 30% changes and p value under 0.1.

#### Quantification and Statistical Analysis

All statistical analyses were performed using two-tailed student T-test. $P < 0.05$ was considered statistically significant. All values are reported as mean ± SEM in each figure.

### RESOURCES TABLE

| Cell lines | | |
|---|---|---|
| **Reagent or Resource** | **Source** | **Identifier** |
| Rat1 fibroblasts | Dr. John Sedivy (Brown University) | N/A |
| ARPE-19 | Sandra Schmid lab | N/A |
| DLD1 | ATCC | CCL-221 |
| LN229 | ATCC | CCL-2611 |
| U-87 MG | ATCC | HTB-14 |
| GBM9 | Dr. Sandeep Burhma (UT, San Antonio) | |
| SF188 | Sigma | SCC282 |
| | | |
| **Plasmids** | | |
| **Reagent or Resource** | **Source** | **Identifier** |
| ARNT2 CRISPR/Cas9 KO Plasmid (h) | Santa Cruz | sc-403101 |
| pIRESpuro-GFP-ARNT2 | This study | N/A |
| pIRESneo-ARNT2 | This study | N/A |
| | | |
| **Antibodies** | | |
| **Reagent or Resource** | **Source** | **Identifier** |
| ARNT2 | Proteintech | 12810-1-AP |
| MYC | Abcam | ab32072 |
| AHR | Enzo Life Sciences | BMLSA2100100 |
| ARNT | Novus Biologic | NB100-110 |
| NPAS4 | Sigma | SAB1402073 |
| SIM2 | MYBIOSOURCE | MBS129140 |
| BMAL | Cell Signaling | 14020 |
| CLOCK | Cell Signaling | 5157 |
| EZH1 | Cell Signaling | 42088 |
| EZH2 | Cell Signaling | 5246 |
| SUZ12 | Cell Signaling | 3737 |
| Histone H3 | Cell Signaling | 4499 |
| Histone H3 (tri methyl K27) | Abcam | ab6002 |
| AHRR | Abcam | ab108518 |
| MAX | Santa Cruz | sc-197 |
| MNT | Santa Cruz | sc-769 |
| MYCN | Santa Cruz | SC-142 |
| Nestin | Santa Cruz | sc-23927 |
| GFAP | Cell Signaling | 12389 |
| Acetylated Tubulin | Sigma | T7451 |
| α-Tubulin | Sigma | T6199 |
| Phospho-p70 S6 Kinase (Thr389) | Cell Signaling | 9234 |
| p70 S6 Kinase | Cell Signaling | 2708 |
| Phospho-mTOR (Ser2448) | Cell Signaling | 5536 |
| mTOR | Cell Signaling | 2983 |
| Cyclin A1 | Novus Biologic | MAB7046 |
| p27 Kip1 | Cell Signaling | 3686 |
| β3-Tubulin | Cell Signaling | 5568 |

| | | |
|---|---|---|
| UCHL1 | Cell Signaling | 13179 |
| Sox2 | Cell Signaling | 3579 |
| Oct-4A | Cell Signaling | 2840 |
| Neurofilament-L | Cell Signaling | 2837 |
| Synaptophysin | Cell Signaling | 36406 |
| β-Actin | Cell Signaling | 4970 |
| | | |
| **Chemicals** | | |
| **Reagent or Resource** | **Source** | **Identifier** |
| UNC1999 | Sigma | 5050520001 |
| GSK126 | Sigma | 5005800001 |
| | | |
| **Oligonucleotides for qPCR** | | |
| **Gene** | **F sequence** | **R sequence** |
| Human ARNT2 | gcctatcctctccgagca | ccaggtatgtctgaagccattt |
| Human MYC | caccagcagcgactctga | gatccagactctgaccttttgc |
| Rat ARNT2 | gaggatcagagccacctacg | gcacttggcccttcagttta |
| Rat MYC | gtgctgcatgaagagacacc | tcaatttcttcctcatcatcttgt |
| Rat AHR | cttcagatgccggctgag | cctcccttggaaattcattg |
| Rat ARNT | ccactgcacaggttacatcaa | tcatcatctgggagggagac |
| 18S rRNA | accgcagctaggaataatgga | gcctcagttccgaaaacca |
| | | |
| **siRNA oligonucleotides** | | |
| **Reagent or Resource** | **Source** | **Identifier** |
| Universal controls #2 | Sigma mission | SIC002 |
| siARNT2-1 | Sigma Mission | SASI_Hs01_00078252 |
| siARNT2-2 | Sigma Mission | SASI_Hs01_00078253 |
| siARNT2-3 | Sigma Mission | SASI_Hs01_00078254 |
| siARNT2-4 | Sigma Mission | SASI_Hs02_00346447 |
| siMYC-3 | Sigma Mission | SASI_Hs01_00222678 |
| siMYC-4 | Sigma Mission | SASI_Hs01_00222680 |
| siEZH1 | Sigma Mission | SASI_Hs02_00332978 |
| siEZH2 | Sigma Mission | SASI_Hs01_00078252 |
| siSUZ12-1 | Sigma Mission | SASI_Hs02_00347682 |
| siSUZ12-2 | Sigma Mission | SASI_Hs01_00107270 |
| siAHR-1 | Sigma Mission | SASI_Hs02_00332181 |
| siAHR-2 | Sigma Mission | SASI_Hs01_00140198 |
| siAHR-4 | Sigma Mission | SASI_Hs01_00140199 |
| siAHRR-1 | GUU UGG AAG UAC AGA GAA A | N/A |
| siAHRR-2 | CAG CAA CGA UCG UGG ACU A | N/A |
| siAHRR-3 | UCA AAU AUA AGG UGG GAA U | N/A |
| siAHRR-4 | GGG ACA AGA CAG ACA AGU A | N/A |
| siMLX-1 | Sigma Mission | SASI_Hs01_00163686 |
| siMLX-2 | Sigma Mission | SASI_Hs01_00163687 |
| siMAX-1 | Sigma Mission | SASI_Hs01_00011941 |
| siMAX-3 | Sigma Mission | SASI_Hs01_00011942 |

| | | |
|---|---|---|
| siMNT-1 | Sigma Mission | SASI_Hs02_00353224 |
| siMNT-2 | Sigma Mission | SASI_Hs01_00094368 |
| | | |
| **Databases** | | |
| **Reagent or Resource** | **Source** | **Identifier** |
| RNA-seq Rat1 fibroblasts MYC OE | Maralice Conacci-Sorrell lab | Lafita-Navarro et al., 2018 |
| RNA-seq LN229 ARNT2 KO | Maralice Conacci-Sorrell lab | GSE236486 |
| | | |
| **Software** | | |
| **Reagent or Resource** | **Source** | **Identifier** |
| FIJI | Image J software | N/A |
| Prism 10 | GraphPad | N/A |
| | | |
| **Other** | | |
| **Reagent or Resource** | **Source** | **Identifier** |
| ChemiDoc Imaging System | BIO-RAD | 17001401 |
| CFX96 Touch Real-Time PCR Detection System | BIO-RAD | 1855195 |
| Zeiss LSM780 Inverted confocal microscope | Zeiss | N/A |
| | | |

## Figures

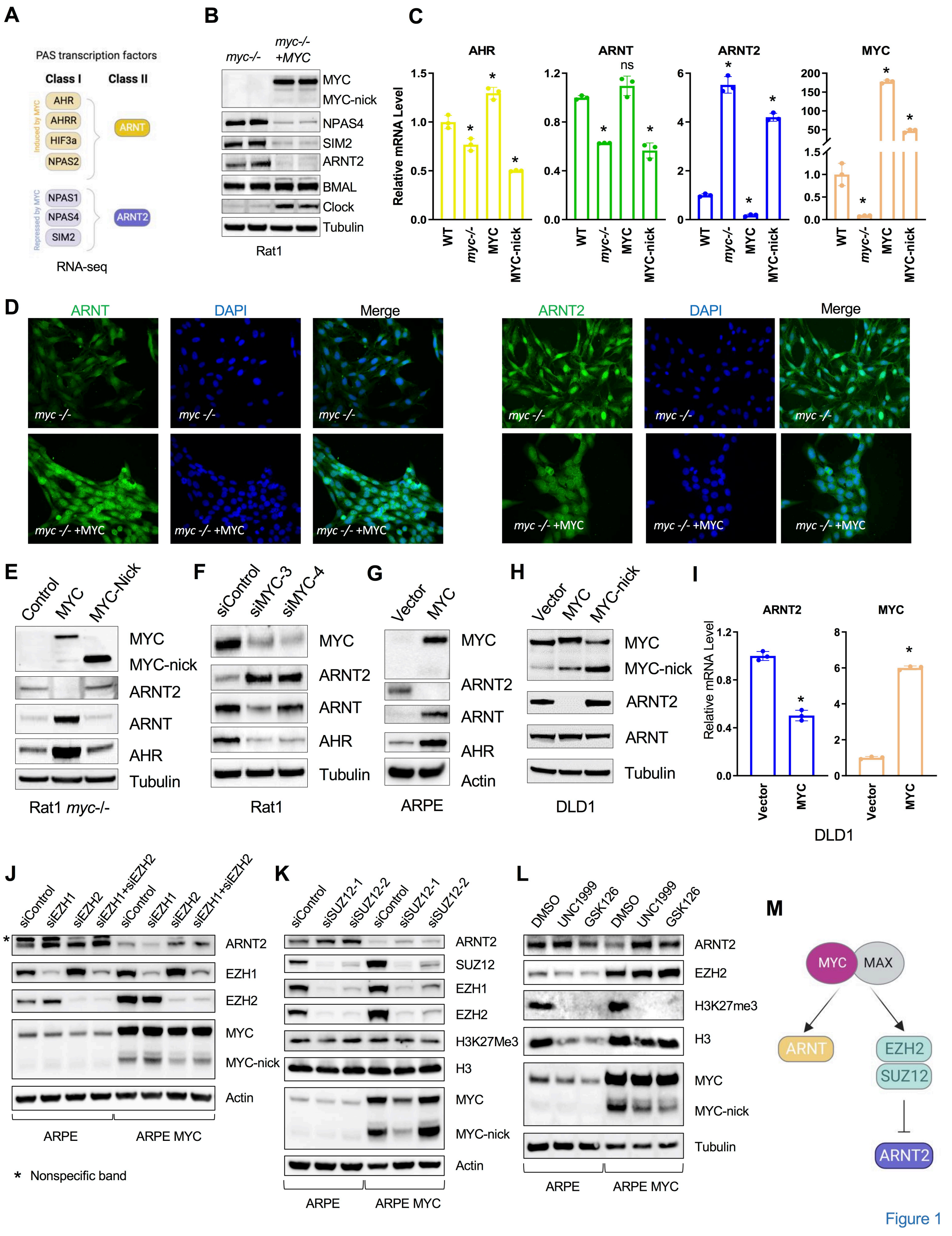

### Figure 1

**Repression of ARNT2 expression by MYC is mediated by the polycomb repressive complex 2.** (A) bHLH-PAS domain transcription factors are regulated by MYC in Rat1 fibroblast RNA-seq dataset (also see Figure S1A). Yellow: upregulated by MYC; Blue: downregulated by MYC. (B) Rat1 fibroblasts *myc-/-* or *myc-/-* reconstituted with human MYC were extracted with NP40 lysing buffer, and the total cell lysates were immunoblotted with the indicated antibodies. (C) RT-qPCR for the indicated genes in Rat1 fibroblasts with different MYC expression profiles. Expression levels of each gene were normalized to the levels of 18S, and the expression levels of each gene in wild-type cells were set to 1. (D) Immunofluorescence of ARNT or ARNT2 in Rat1 fibroblasts *myc-/-* or *myc-/-* reconstituted with human MYC. Nuclei were stained with DAPI. (E) Rat1 fibroblasts *myc-/-*or *myc-/-* reconstituted with human MYC or MYC-nick were extracted with NP40 lysing buffer, and the total cell lysates were immunoblotted with the indicated antibodies. (F) Rat1 fibroblasts were transfected with control or MYC siRNAs, and total cell lysates were immunoblotted with the indicated antibodies. (G) Total cell lysates of human epithelial ARPE-19 cell expressing empty vector or MYC were immunoblotted with the indicated antibodies. (H) Total cell lysates of human colon cancer cell DLD1 expressing empty vector, MYC, or MYC-nick were immunoblotted with the indicated antibodies. (I) RT-qPCR for ARNT2 and MYC in DLD1 cells expressing empty vector or MYC. Expression levels of each gene were normalized to the levels of 18S, and the expression levels in vector cells were set to 1. (J) and (K) ARPE-19 cells expressing vector or MYC were transfected with control siRNA or siRNAs targeting PRC2 genes, and total cell lysates were immunoblotted with the indicated antibodies. (L) ARPE-19 cells expressing vector or MYC were treated with DMSO, 5 μM UNC1999, or 5 μM GSK126 for 3 days, and total cell lysates were immunoblotted with the indicated antibodies. (M) Model of mechanism demonstrating how PRC2 regulates ARNT2 expression.

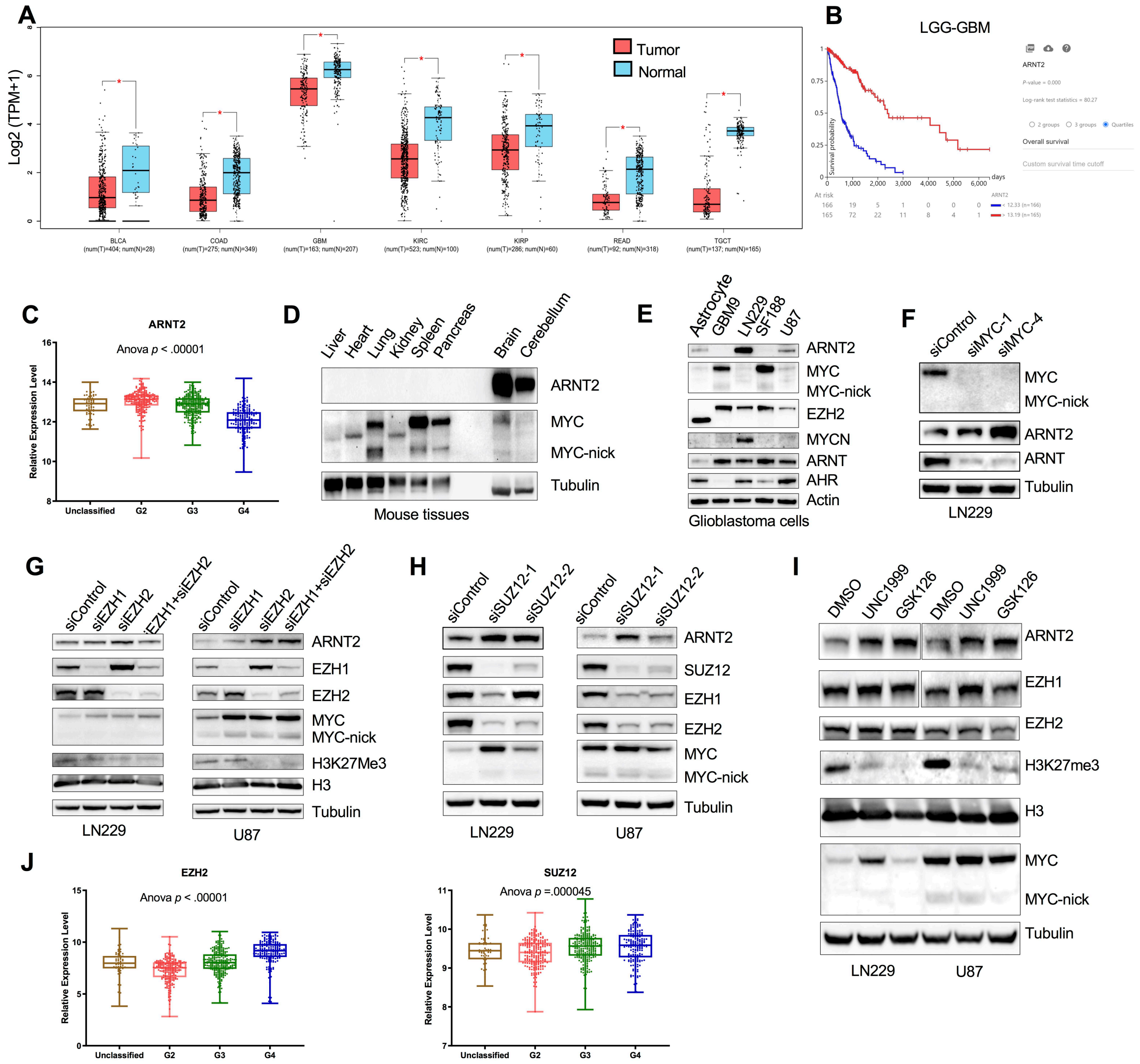

Figure 2

### Figure 2

**ARNT2 is highly expressed in brain and cerebellum and repressed in GBM patients and cells**. (A) ARNT2 mRNA expression in tumor and normal tissues from patients of different tumor types deposited in TCGA and GTEx, and analyzed by Gene Expression Profiling Interactive Analysis (GEPIA) web server. |Log2FC| cutoff 0.585, and p-value cutoff 0.01. (B) Kaplan–Meier curve comparing survival of glioma (LGG-GBM) patients with the highest and the lowest levels of ARNT2. Comparison between patients with the 25%

highest and the 25% lowest ARNT2 expression were generated using Xena Functional Genomics Explorer (https://xenabrowser.net/). (C) ARNT2 gene expression levels in glioma grades. Data are from TCGA. (D) Mouse tissues were extracted with NP40 lysis buffer, and total lysates were immunoblotted with the indicated antibodies. (E) Astrocytes and glioblastoma cells were extracted with NP40 lysis buffer, and total cell lysates were immunoblotted with the indicated antibodies. (F) Glioblastoma cells LN229 were transfected with control or MYC siRNAs, and total cell lysates were immunoblotted with the indicated antibodies. (G) and (H) Glioblastoma cells LN229 and U87 were transfected with control siRNA or siRNAs targeting PRC2 genes, and total cell lysates were immunoblotted with the indicated antibodies. (I) LN229 and U87 cells were treated with DMSO, 5 μM UNC1999, or 5 μM GSK126 for 3 days, and total cell lysates were immunoblotted with the indicated antibodies. (J) EZH2 and SUZ12 gene expression levels in glioma grades. Data are from TCGA.

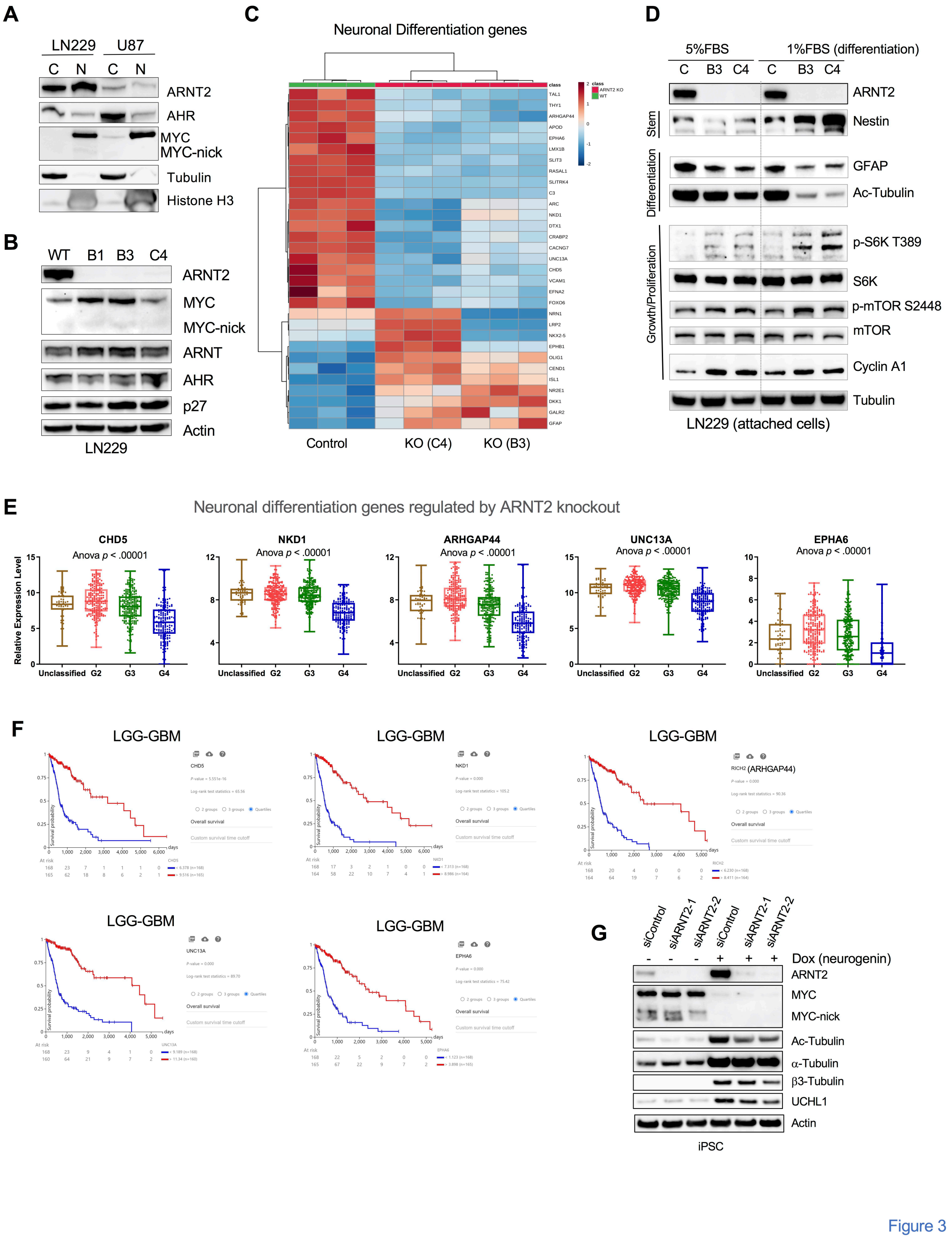

### Figure 3

**Knocking out ARNT2 from GBM cells decreases the expression of neuronal differentiation markers and increases the expression of stem cell markers**. (A) LN229 and U87 cells were fractionated into cytosolic and nuclear fractions, and the samples were immunoblotted with the indicated antibodies. (B) Single clones of ARNT2 CRISPR knockout (KO) cells were selected, extracted, and immunoblotted with the indicated antibodies. (C) Wild-type (WT) and ARNT2 CRISPR KO LN229 cells were collected and subjected to RNA-seq analysis. The heatmap was generated based on relative expression level of genes involving neuronal differentiation with cutoff of Log2FC=0.585 in both KO clones. (D) Wild-type and ARNT2 KO LN229 cells were cultured in normal 5% FBS or 1% FBS for 20 days to induce differentiation and then were extracted for immunoblotting. (E) Expression levels of neuronal differentiation genes regulated by ARNT2 KO in glioma grades. Data are from TCGA. (F) Kaplan–Meier curve comparing survival of patients with glioma (LGG-GBM) patients with the highest and the lowest levels of the same genes. Comparisons between patients with the 25% highest and 25% lowest gene expression were generated using Xena Functional Genomics Explorer (https://xenabrowser.net/). (G) iPSCs were transfected with control and ARNT2 siRNA before induction to differentiate. Final cells were extracted for immunoblotting.

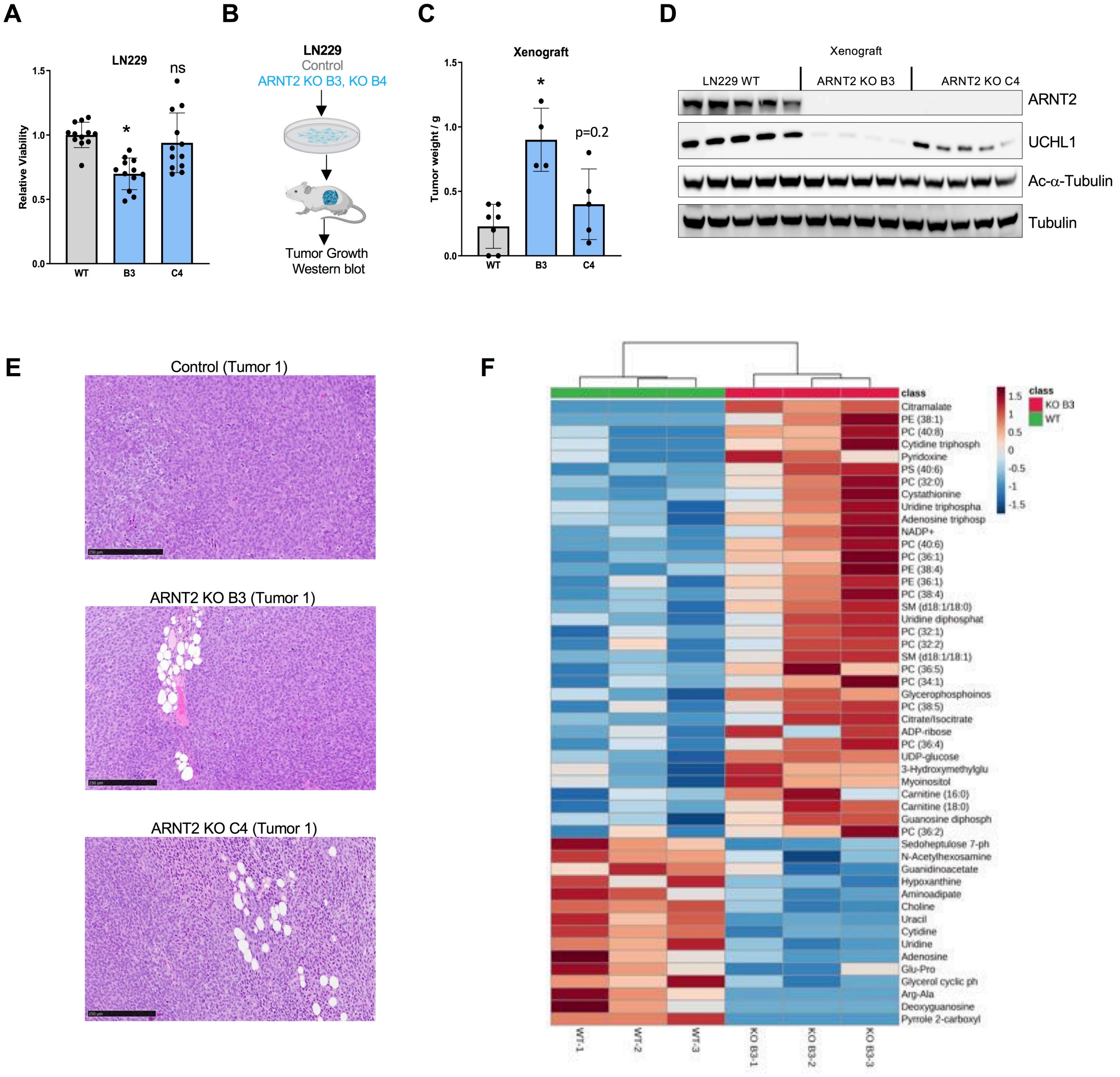

Figure 4

### Figure 4

**Knocking out ARNT2 increases the growth of GBM cells in vivo and ARNT2 KO tumors display an increase in lipid droplets and phospholipids content.** (A) Aggregate of multiple cell proliferation assays between wild-type (WT) and ARNT2 KO LN229 cells. (B) Diagram of xenograft study of LN229 cells. (C) Tumor weight of xenograft of WT and ARNT2 KO LN229 cells. (D) Xenograft tumors were extracted with NP40 lysis buffer, and total lysates were immunoblotted with the indicated antibodies. (E) Xenograft tumors were fixed with paraformaldehyde and stained with hematoxylin and eosin (H&E). (F) Xenograft tumors were extracted with 80% methanol and submitted for metabolomics assay. Metabolites with at least 30% change and p under 0.1 were plotted in the heatmap.

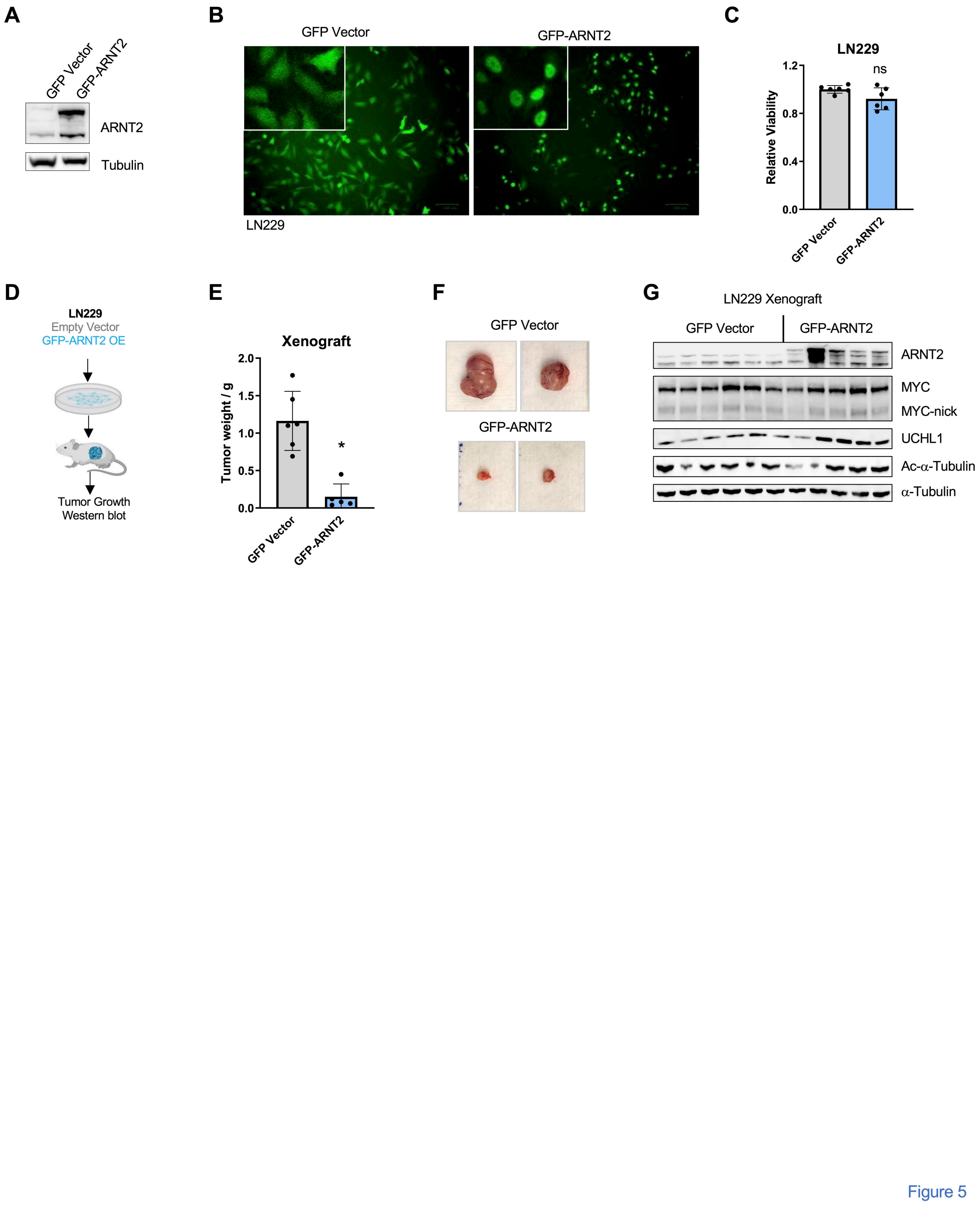

### Figure 5

**Overexpression of ARNT2 prevents tumor growth**. (A) LN229 stably expressing empty vector (GFP vector) or GFP-tagged ARNT2 were extracted with NP40 lysis buffer, and total cell lysates were immunoblotted with the indicated antibodies. (B) Fluorescence microscopic pictures of LN229 stably expressing GFP vector or GFP-tagged ARNT2. (C) Cell proliferation assays LN229 stably expressing GFP vector or GFP-tagged ARNT2. (D) Diagram of xenograft study of LN229 ARNT2 overexpressing cells. (E) Tumor weight of xenograft of LN229 stably expressing GFP vector or GFP-tagged ARNT2. (F) Pictures of selected tumors. For pictures of all tumors see Figure S5C. (G) Xenograft tumors were extracted with NP40 lysis buffer and total lysates were immunoblotted with the indicated antibodies.

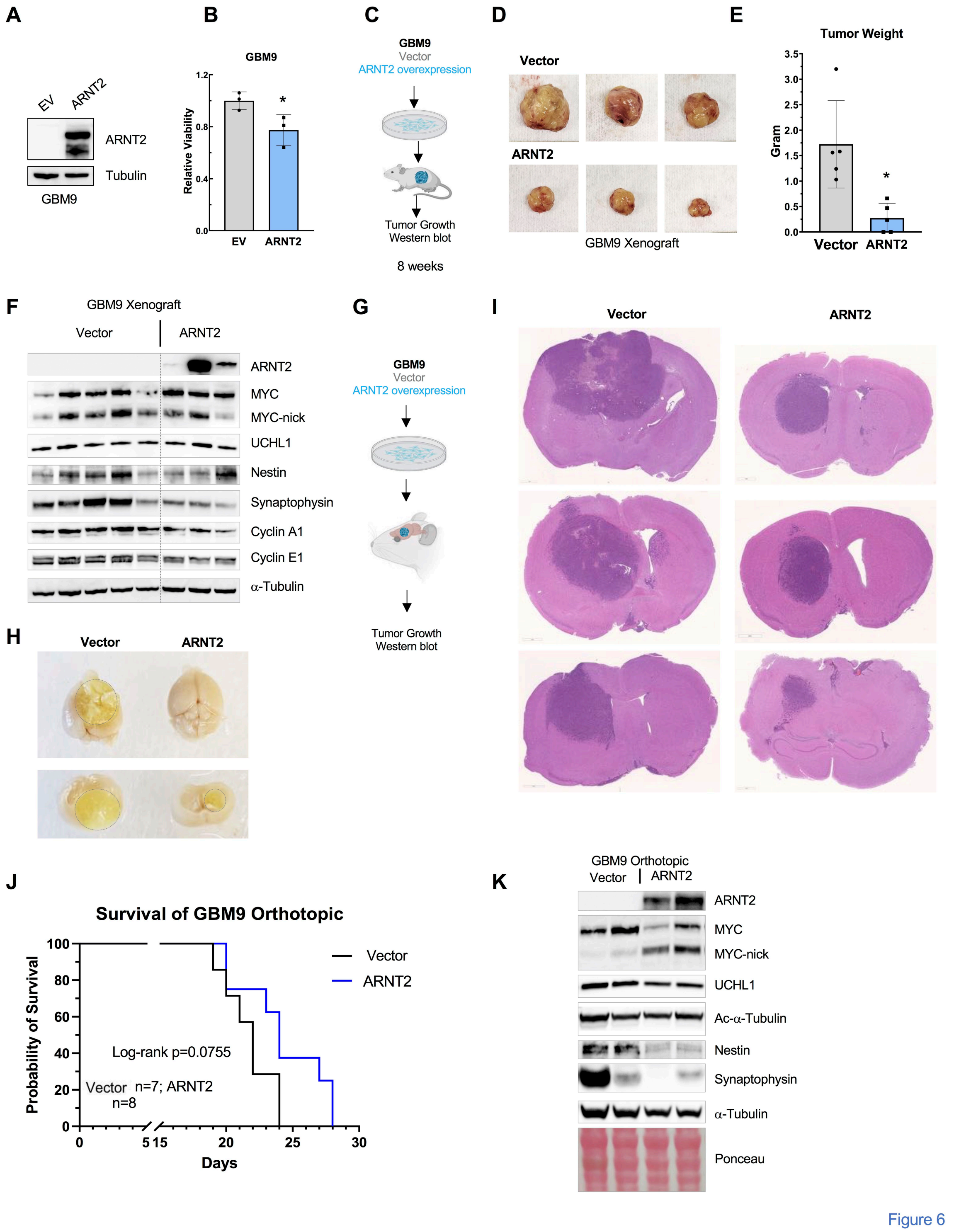

Figure 6

### Figure 6

**Ectopic expression of ARNT2 reduced the growth of orthotopically xenografted GBM cells. (**A) GBM9 stably expressing empty vector or ARNT2 were extracted with NP40 lysis buffer, and total cell lysates were immunoblotted with the indicated antibodies. (B) Cell proliferation assays GBM9 stably expressing empty vector or ARNT2. (C) Diagram of xenograft study of GBM9 cells. (D) Pictures of tumors. (E) Tumor weight of xenograft of GBM9 stably expressing empty vector or ARNT2. (F) Xenograft tumors were extracted with NP40 lysis buffer and total lysates were immunoblotted with the indicated antibodies. (G) Diagram of orthotopic xenograft study of GBM9 cells. (H) Pictures of the brains. The color of the tumors comes from GFP expression in GBM9 cells. (I) Brains and tumors were fixed with paraformaldehyde and stained with hematoxylin and eosin (H&E). (J) Kaplan–Meier curve comparing survival of mice injecting of GBM9 cells expressing empty vector or ARNT2. (K) Isolated tumors were extracted with NP40 lysis buffer and total lysates were immunoblotted with the indicated antibodies.

### Supplementary Files

This is a list of supplementary files associated with this preprint. Click to download.

- FigureS1.jpg
- FigureS2.jpg
- FigureS3.jpg
- FigureS4.jpg
- FigureS5.jpg
- FigureS6.jpg
